# Case studies in health supply chain workforce management

**DOI:** 10.1186/2052-3211-7-S1-O9

**Published:** 2014-12-17

**Authors:** Taylor Wilkerson

**Affiliations:** 1 Logistics Management Institute (LMI), McLean, Virginia, USA

## Background

Logistics Management Institute (LMI) supports People that Deliver in developing case studies to illustrate how supply chain organizations manage their personnel and workforce. The intent of these case studies is to serve as a guide for other organizations in managing supply chain personnel effectively to improve supply chain performance.

## Method

LMI created a questionnaire to cover the five building blocks of workforce development: engaged stakeholders, optimise policies and plans, workforce development, increase performance, professionalization of SCM (USAID|DELIVER 2013). In collaboration with PtD, we then contacted potential case study participants and, when agreed, conducted an interview using the structured questionnaire. LMI also collected relevant documents from the case study participants.

## Results

Case study interviews have been conducted with two organizations to date: Sudan Central Medical Stores (CMS) and Imperial Health Sciences (IHS). Those case studies have been documented and compared, demonstrating a comprehensive approach to workforce management, including recruiting, performance management, training and development, and professionalization. The results illustrate differing focuses between the two organizations, with the Sudan CMS focusing more on meeting stakeholder objectives and IHS focusing more on workforce design and financial factors. LMI and PtD are working to add additional case studies to this series.

## Discussion

The two studies illustrate the distinction between public and private sector workforce management. These case studies serve as examples of effective workforce management for others to review to identify practices that can improve their supply chain workforce. Both Sudan CMS and IHS use good workforce management practices; however, there is still room for improvement. Both organizations demonstrate that effective workforce management in health supply chains can be achieved with the right leadership and resources. The result is improved supply chain reliability and cost performance.

## Lessons learned

Effective workforce management is essential for health supply chain success. Organizations that tailor workforce management practices to the needs of their supply chain develop more competent and knowledgeable staff. The result is a more effective supply chain operation that maintains quality, cost, and service with reduced management burdens.

